# Differences in the prevalence and clinical correlates of comorbid suicide attempts in patients with early- and late-onset major depressive disorder

**DOI:** 10.3389/fpsyt.2023.1173917

**Published:** 2023-08-01

**Authors:** Xiao Huang, Yuan Sun, Anshi Wu, Xiangyang Zhang

**Affiliations:** ^1^Department of Anesthesiology, Beijing Chao-Yang Hospital, Capital Medical University, Beijing, China; ^2^Department of Pharmacy, Beijing Chao-Yang Hospital, Capital Medical University, Beijing, China; ^3^CAS Key Laboratory of Mental Health, Institute of Psychology, Beijing, China; ^4^Department of Psychology, University of Chinese Academy of Sciences, Beijing, China

**Keywords:** MDD (major depressive disorder), suicide attempts, age at onset, prevalence, outpatients

## Abstract

**Objective:**

There are many studies on differences in the onset age of major depressive disorder (MDD) patients. However, study on differences in clinical correlates of suicide attempts between early- and late-onset MDD patients is limited. The aim of this study was to investigate the differences in the prevalence and clinical correlates of suicide attempts in patients with early- and late-onset MDD in China.

**Methods:**

A total of 1718 adult outpatients with MDD were recruited. Demographic and clinical data were collected. The 17-item Hamilton Rating Scale for Depression (HAMD-17), Hamilton Anxiety Rating Scale (HAMA), Positive and Negative Syndrome Scale (PANSS) positive subscale, and Clinical Global Impression-Severity (CGI-S) Scales were used to assess their depressive, anxiety, psychotic symptoms, and the severity of the clinical symptoms, respectively.

**Results:**

The prevalence of suicide attempts was higher in late-onset MDD patients (291/1369, 21.3%) than in early-onset MDD patients (55/349, 15.8%) (*p* = 0.023). However after Bonferroni correction no significant difference was found in the prevalence of suicide attempts in late-onset and late-onset MDD patients (*p* > 0.05). In both early- and late-onset groups, univariate analysis showed that the following characteristics were significantly associated with suicide attempts: HAMA, HAMD and PANSS positive subscale scores, thyroid stimulating hormone (TSH) levels, blood glucose levels, systolic blood pressure (SBP), and diastolic blood pressure (DBP). In both the early- and late-onset groups, the prevalence rates of severe anxiety disorder and psychotic symptoms were significantly higher in the suicide attempt group than in the non-suicide attempt group. In regression analysis, disease duration, TSH levels and HAMA score were independently associated with suicide attempts in the early-onset group, while TSH levels, HAMA and HAMD score were independently associated with suicide attempts in the late-onset group.

**Conclusion:**

This study suggests that suicide attempts are not frequent in early-onset outpatients with MDD compared with late-onset, and some clinical correlates are associated with suicide attempt in early- and late-onset MDD.

## 1. Introduction

Depression is one of the most prevalent disabling illnesses in the world, with an estimated 264 million people suffering from it worldwide (1). Major depressive disorder (MDD) is one of the leading causes of disability contributing to the global burden of disease. MDD is marked by signs and symptoms such as depressed feelings that persist for more than 2 weeks and lead to emotional distress, health issues, and suicide (2). In China, depression is estimated to be the second leading cause of disability in terms of life expectancy (3). The total lifetime prevalence of depression in the Chinese population is approximately 6.9% (4).

Suicide is a serious public health problem. In recent years, the correlation between depression and suicide attempts has received significant attention. Suicide in MDD and other mood disorders might be multidimensional, including several easy treatment, perhaps with different neurobiological underpinnings. For example, homocysteine dysregulation may be associated with suicide ideation in alexithymic MDD outpatients (5). Genetics could also play an important role in affecting an individual’s suicide risk (6). Preventing suicide in personalized and accurate psychiatry is needed (7). Indeed, MDD is the most common psychiatric diagnosis associated with suicide (8). Suicide imposes a heavy burden on society, with nearly 800,000 people dying by suicide each year (9). The results of a meta-analysis by Cai et al. has shown that the overall prevalence of suicidal ideation in MDD patients is 37.7% and the combined prevalence of suicidal planning is 15.1% (10). Additional data show an upward trend in suicide rates compared to the past decades (11). An understanding of the clinical and neuropsychological risk factors associated with suicidal behavior will provide the possibility to prevent such behaviors in patients with MDD in clinical practice.

Based on the time of onset, MDD patients can be classified as early-onset or late-onset groups. Whether early-onset MDD patients have a higher incidence of suicidal behavior than late-onset MDD patients remains controversial. However, early-onset MDD patients have more severe illness, psychosis, and suicidal ideation than late-onset MDD patients. For example, childhood-onset MDD is associated with a higher rate of serious cardiovascular events compared to adult-onset MDD (12). A study by Zisook et al. showed that compared to late-onset MDD, patients with early-onset MDD have a higher burden of illness, including more impaired social and occupational functioning, poorer quality of life, a more negative view of life, suicide attempts and suicidal ideation (13). Compared to early-onset patients with MDD, late-onset MDD patients were less likely to have a history of self-harm or to take psychotropic medications (14). Patients with early-onset MDD more frequently suffered from recurrent depression and were reported to have suicidal ideation compared to those with moderate-onset and late-onset MDD patients. Xiao et al. also found that early-onset MDD patients had more severe symptoms, negative views of self, suicidal ideation, and restlessness compared to late-onset MDD patients (15). In Chinese Han women with MDD, early-onset patients were associated with more suicidal ideation and attempts and higher levels of neuroticism than late-onset patients (16). However, Chan et al. found that MDD patients with an onset age older than 50 years were at higher risk for subsequent Parkinson’s disease (17). In addition, a previous study by Herzog et al. showed that the outcome of antidepressant treatment for MDD patients depended on their age of onset, and early-onset patients report more often suicidal ideation compared with intermediate and late-onset MDD patients (18). Park et al. found that early-onset depression was related with chronic (recurrent and longer episode) and severe (higher lifetime suicidality) clinical features among korean adults aged 40 and older MDD (19).

Studies on differences in clinical correlates of suicide attempts between early- and late-onset MDD patients are still limited, especially in Han Chinese population. Therefore, using a large sample of first-episode, untreated Chinese MDD patients, we aimed to compare the differences in the prevalence and clinical correlates of suicide attempts between early- and late-onset MDD patients. We hypothesized that early-onset MDD patients would have a higher prevalence and more severe clinical symptoms than late-onset MDD patients.

## 2. Methods

### 2.1. Design and participants

This cross-sectional study was conducted at the First Hospital of Shanxi Medical University, Taiyuan, China during 2015–2017. The study was approved by the Ethics Committee of the hospital (No. 2016-Y27). All participants signed an informed consent form before participating in the study. Eligibility criteria included: (1) MDD diagnosed by two independent psychiatrists based on the Structured Clinical Interview for DSM-IV (SCID), (2) first episode, not receiving any medication before, (3) male or female patients aged 18–60 years, Han Chinese, (4) willing to participate in the study, and (5) able to understand the instructions of clinical psychiatrists. A total of 1796 individuals were screened. 78 patients were excluded for the following reasons: (1) severe physical illness and severe personality disorder (*n* = 24); (2) substance abuse and dependence, including alcohol consumption but not smoking (*n* = 9); (3) female patients who were pregnant or breastfeeding (*n* = 10); (4) unable to be interviewed for an acute clinical condition (*n* = 5), (5) unwillingness to provide a written consent form (*n* = 21) and other unspecified reasons (n = 9). Finally a total of 1718 outpatients were recruited from the Department of Psychiatry of this hospital.

### 2.2. Sociodemographic information and clinical measures

Sociodemographic information was collected for each patient, including age, gender, marital status, age at onset of MDD, duration of disease, and education level.

Clinical information included body weight and height, systolic blood pressure (SBP), and diastolic blood pressure (DBP), which were collected by trained investigators. The calculation of BMI is weight divided by the square of height. Based on previous studies, clinical experience and the age distribution of the included patients to involve a sufficient sample size of subgroups to have statistical power, 22 years was used as the MDD onset age cut-off value in this study (early onset, <22 years; late onset, ≥22 years) (20, 21).

In the present study, the symptoms of anxiety, depression, psychosis, and the severity of illness were assessed by the Hamilton Anxiety Rating Scale (HAMA), 17-item Hamilton Rating Scale for Depression (HAMD-17), Positive and Negative Syndrome Scale (PANSS) positive subscale, and Clinical Global Impression-Severity (CGI-S), respectively. The maximum score of the HAMA is 56 with 14-point scale. The score of 18–24 is considered mild to moderate severity and 25–30 is considered as moderate to severe (22). The HAMD-17 consisted of eight items that are scored on a 5-point scale, ranging from 0 (none) to 2 (symptom-specific severity), with higher scores indicating more severe depressive symptoms (23). Psychotic symptoms were assessed by the Positive and Negative Syndrome Scale (PANSS) positive subscale. The score consists of 7 items that scored on a 7-point scale, ranging from 1 (non-existent) to 7 (extremely severe). Patients with a total positive symptom score ≥ 15 were identified as having psychotic symptoms (24).

A suicide attempt is an attempt by an individual to self-harm in some way to end his or her life. Participants were asked: “Have you ever attempted suicide in your life?” If the response was yes. He/she was categorized as a suicide attempter. Else, he/she was a non-suicide attempter. We also inquired for specific details about their suicide attempts, including the number, timing, and method of attempts, the method of the suicide attempt. If the patient could not provide a definitive answer, we performed additional interviews with family members or friends to obtain clarification of this information.

The above information was obtained from two experienced psychiatrists with special training. The internal consistency coefficients of the HAMD, HAMA and PANSS total scores were 0.85, 0.84 and 0.82, respectively.

### 2.3. Measurement of thyroid function, lipid profile, and fasting glucose

Biochemical indicators, including fasting blood glucose, thyroid stimulating hormone (TSH), free triiodothyronine 3 (FT3) and free thyroxine 4 (FT4), were obtained for each patient under fasting conditions (between 6 and 8 am). Serum levels of TSH, FT3 and FT4 were measured by electrochemiluminescence immunoassay (Roche Diagnostics, Indianapolis, IN, United States). The glucose oxidase method was used to test fasting blood glucose levels. Enzymatic colorimetric assay was used to test the lipid profile.

### 2.4. Statistical analysis category

The data were analyzed by using SPSS version 26.0. The distribution of the data was tested by Kolmogorov–Smirnov (KS) two-sample test. Normally distributed variables were compared between the two groups by ANOVA, and non-normally distributed data were compared by the Mann–Whitney U test. Chi-square tests were performed to compare the categorical variables. Bonferroni correction was used for multiple testing. Binary regression analyses were performed to explore factors independently associated with suicide attempts. In logistic regression analysis, suicide attempts in each onset age group were the dependent variable, whereas variables that differed significantly between the groups with and without suicide attempts in univariate analysis were independent variables. Area under receiver operating characteristic (AUCROC) was used to determine the ability of important variables to discriminate between early-onset and late-onset MDD patients with and without suicide attempts. A consistency statistic >0.7 was generally considered acceptable (25, 26). A two-tailed *p* value of 0.05 was set for significance.

## 3. Results

### 3.1. Comparison of the prevalence of suicide attempts of MDD patients in terms of age at onset

A total of 1718 patients were included in the statistics (49 patients of age < 22 years, 344 patients of age 22–29 years, 377 patients of age 30–39 years, 395 patients of age 40–49 years, and 253 patients of age > 49 years). There were 349 patients included in early-onset group (age < 22 years) and 1,369 patients included in late-onset group (age≧22). Patients in the early-onset group reported fewer suicide attempts (55/349, 15.8%) than those in the late-onset group (291/1369, 21.3%; *p* = 0.023, OR = 1.443, 95% CI = 1.052–1.979). After Bonferroni correction, no significant difference was found in the suicide attempt rate (*p* > 0.05). MDD patients in the late-onset group were older, had a longer disease duration, higher SBP, higher DBP, and higher rates of married status compared to those in the early-onset group (all *p* < 0.05).

### 3.2. Comparison of suicide attempts In MDD patients in terms of age at onset

In both early- and late-onset groups, univariate analysis (Table 1) showed that the following characteristics were significantly associated with suicide attempts: HAMA, HAMD and PANSS positive subscale scores, TSH levels, blood glucose levels, SBP and DBP (all *p* < 0.05). In both the early- and late-onset groups, the prevalence rates of severe anxiety disorder and psychotic symptoms were significantly higher in the suicide attempt group than in the non-suicide attempt group (both *p* < 0.001). In the late-onset group, the duration of illness was significantly longer in the suicide attempt patients than in the non-suicide attempt group, however it did not remained after Bonferroni correction (*F* = −2.064, *p* = 0.04; Bonferroni corrected *p* > 0.05).

**Table 1:** Differences in sample characteristics by suicide attempts and age of onset in MDD.

	Early onset (*n* = 349)				Late onset (*n* = 1,369)			
	With suicide attempts (*n* = 55)	Without suicide attempts (*n* = 294)	*F*	*P*	With suicide attempts (*n* = 291)	Without suicide attempts (*n* = 1,078)	*F*	*p*
Actual age, year	19 (18,20)	19 (18,20)	−1.633	0.107	38(30.5,48)	39 (29,47)	−0.716	0.474
Duration of disease, month	3.5 (2.5,6)	3.5 (2.5,5)	−1.153	0.253	6(4,9)	5.5 (3,8.75)	−2.064	0.04
Sex, *n* (%)			0.085	0.771			0.159	0.69
Male	22 (40)	127(43.2)			90 (30.93)	349 (32.37)		
Female	33 (60)	167(56.8)			201 (69.07)	729 (67.63)		
Married, *n* (%)	5 (9.1)	27(9.2)	0.001	0.983	246 (84.5)	938 (87)	1.0001	0.317
Education level, *n* (%)			0.645	0.885			5.592	0.133
1	2 (3.6)	8 (2.7)			98 (33.7)	305 (28.29)		
2	39 (70.90)	197 (67.0)			102 (35.1)	422 (39.15)		
3	13 (23.6)	81 (27.6)			68 (23.4)	287 (26.62)		
4	1 (1.8)	8 (2.7)			23 (7.9)	64 (5.94)		
TSH	6.95 (4.7,9.23)	4.14 (2.85,5.77)	−6.486	<0.001	6.71 (4.39,8.865)	4.73 (2.9,6.22)	−10.424	<0.001
BMI	23.56 (22.37,25.175)	24.13 (23.14,25.47)	0.931	0.355	24.36 (23.32,26.11)	24.25 (23.25,25.6)	0.053	0.958
T3	4.89 (0.76)	4.97 (0.72)	0.486	0.486	4.91 (0.72)	4.9 (0.7)	0.487	0.485
T4	17.19 (2.94)	16.65 (3.03)	1.489	0.223	16.58 (3.16)	16.7 (3.1)	0.506	0.477
Fating blood glucose (mmol/L)	5.67 (0.79)	5.28 (0.63)	16.73	<0.001	5.57 (0.73)	5.4 (0.6)	23.066	<0.001
SBP, mmHg	116.3 (11.28)	108.71 (8.771)	31.378	<0.001	126.0 (11.5)	120.8 (9.0)	64.311	<0.001
DBP, mmHg	72.69 (5.68)	71.5 (5.95)	16.732	<0.001	79.2 (7.8)	76.3 (6.0)	46.512	<0.001
Severe anxiety, *n* (%)	14 (25.5)	17 (5.8)	19.79	<0.001	97 (33.3)	76 (7.1)	141.012	<0.001
Exhibiting psychotic symptoms, *n* (%)	14 (25.5)	22 (7.5)	14.291	<0.001	74 (25.4)	61 (5.7)	98.556	<0.001
HAMD	32 (31,34)	30 (28,32)	−6.569	<0.001	32 (30,34)	30 (28,32)	−12.519	<0.001
HAMA	23 (21,24.5)	20 (18,22)	−6.007	<0.001	23 (21,27)	20 (18,22)	−15.691	<0.001
PANSS	7 (7,15.5)	7 (7,7)	−3.564	<0.001	8 (7,17.5)	7 (7,7)	−8.422	<0.001

Data expressed as mean ± SD, median (interquartile range), or percentage. Education degree: 1, middle school; 2, high school; 3, college; 4, graduate. TSH, thyroid stimulating hormone; BMI, body mass index; FT3, free triiodothyronine; FT4, free thyroxine; SBP, systolic blood pressure; DBP, diastolic blood pressure; HAMD, Hamilton Rating Scale for Depression; HAMA, Hamilton Anxiety Scale; PANSS, Positive and Negative Syndrome Scale.

### 3.3. Risk factors for suicide attempts in early-onset group

**Figure 1:**
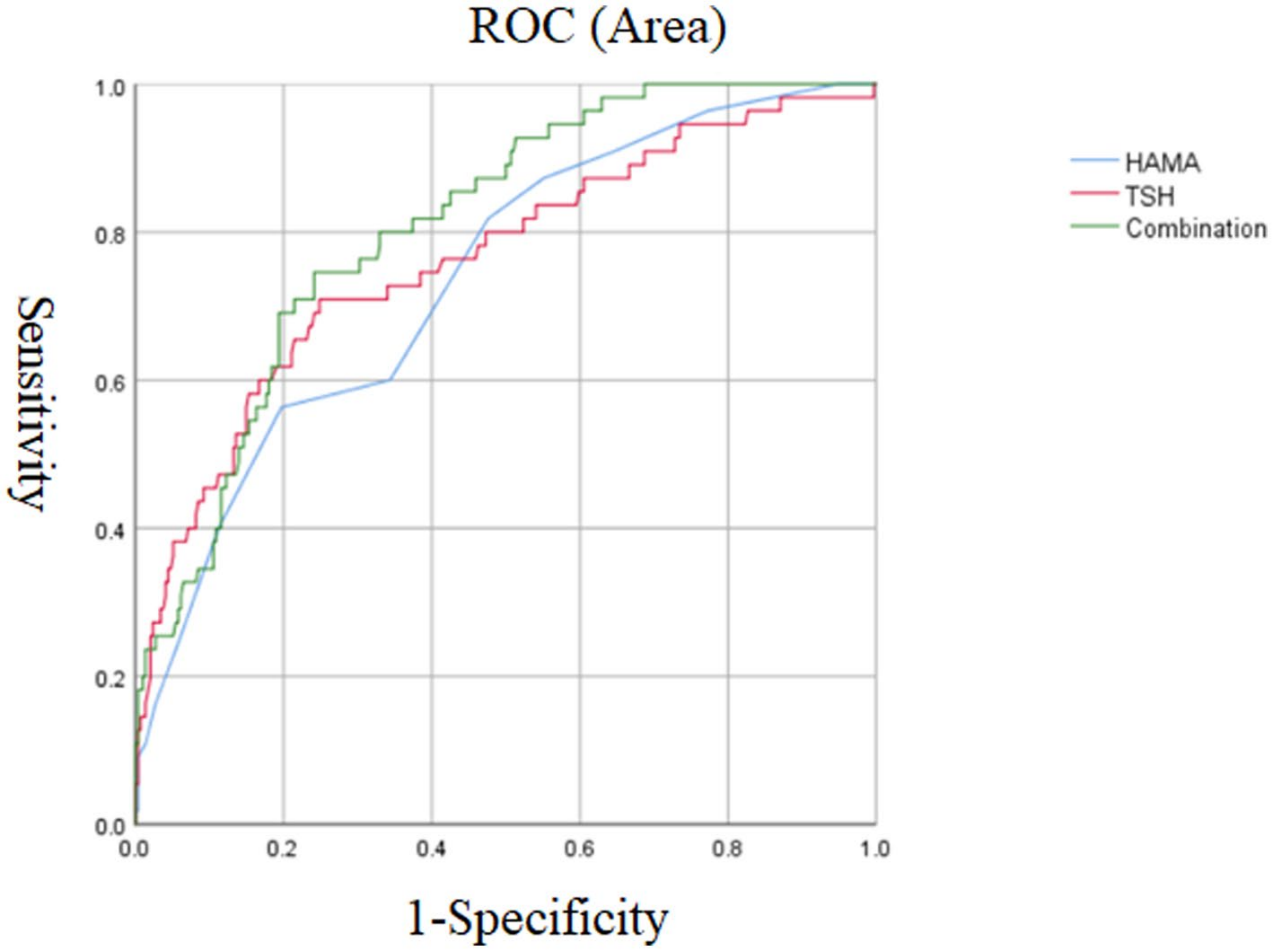
The discriminatory capacity of HAMA score, TSH and the combination of these two factors for distinguishing between patients with and without suicide attempts in early onset MDD. The area under the curve of HAMA score, TSH, and the combination of these two factors were 0.738, 0.763, and 0.808, respectively. ROC: receiver operating characteristic. HAMA: Hamilton Anxiety Scale. TSH: thyroid stimulating hormone. MDD, major depressive disorder.

Logistic regression analysis showed that in the early-onset group, the following variables were independently associated with suicide attempts: the duration of illness (*p* = 0.033, OR = 1.14, 95% CI = 1.01–1.28), TSH level (*p* = 0.006, OR = 1.29, 95% CI = 1.08–1.55) and HAMA score (*p* = 0.009, OR = 1.22, 95% CI = 1.05–1.41). In addition, the AUCROC showed the following values for each risk factor: duration of illness was 0.534, TSH was 0.763, and HAMA was 0.738. When we combined parameters with AUC values ≥0.7, we found that the combination of TSH level and HAMA score had a higher AUC value of 0.808, which could distinguish suicide attempters from non-suicide attempters in MDD patients (*p* < 0.001, 95% CI = 0.751–0.864) (Table 2; Figure 1).

**Table 2:** Risk factors of suicide attempts in MDD with early onset.

	*B*	*W*	*D*	*p*	OR	95%CI lower	95%CI upper
Duration of disease	0.127	4.558	1	0.033	1.136	1.01	1.277
TSH	0.257	7.486	1	0.006	1.293	1.076	1.554
Fating blood glucose	0.213	0.641	1	0.423	1.237	0.735	2.084
SBP	0.016	0.518	1	0.472	1.017	0.972	1.063
HAMD	0.127	2.053	1	0.152	1.135	0.954	1.35
HAMA	0.197	6.845	1	0.009	1.218	1.051	1.411
PANSS	−0.039	0.753	1	0.386	0.962	0.881	1.05

MDD, major depressive disorder; TSH, thyroid stimulating hormone; SBP, systolic blood pressure; HAMD, Hamilton Rating Scale for Depression; HAMA, Hamilton Anxiety Scale; PANSS, Positive and Negative Syndrome Scale.

### 3.4. Risk factors for suicide attempts in late-onset group

**Figure 2:**
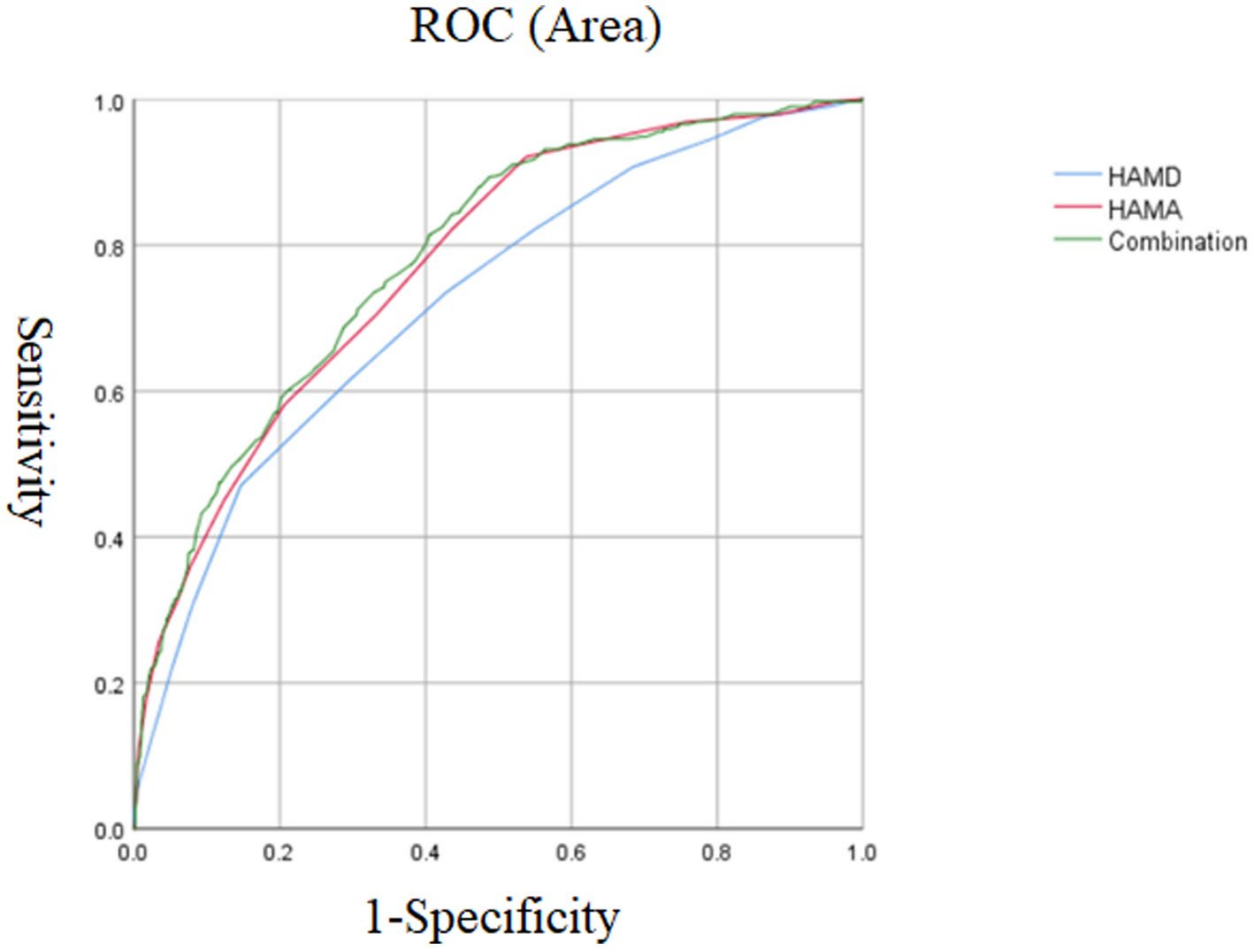
The discriminatory capacity of HAMD score, HAMA score and the combination of these two factors for distinguishing between patients with and without suicide attempts in late onset MDD. The area under the curve of HAMD score, HAMA score, and the combination of these two factors were 0.724, 0.775, and 0.784, respectively. ROC, receiver operating characteristic. HAMD, Hamilton Rating Scale for Depression. HAMA, Hamilton Anxiety Scale. MDD, major depressive disorder.

Logistic regression analysis showed that in the late-onset group, the following variables were independently associated with suicide attempts: TSH level (*p* = 0.001, OR = 1.13, 95%CI = 1.06–1.23), SBP (*p* = 0.017, OR = 1.02, 95%CI = 1.00–1.04), HAMD (*p* = 0.017, OR = 1.09, 95%CI = 1.02–1.17) and HAMA (*p* < 0.001, OR = 1.29, 95%CI = 1.22–1.38). Furthermore, the AUCROC showed the following values for each risk factor: TSH was 0.698, SBP was 0.638, HAMD was 0.724, and HAMA was 0.775. When we combined the parameters with an AUC value ≥0.7, we found that the combination of HAMD and HAMA scores had a higher AUC value of 0.784, which could distinguish suicide attempters from non-suicide attempts in MDD patients (*p* < 0.001, 95% CI = 0.755–0.813) (Table 3; Figure 2).

**Table 3:** Risk factors of suicide attempts in MDD with late onset.

	*B*	*W*	*D*	*p*	OR	95%CI lower	95%CI upper
Duration of disease	−0.004	0.082	1	0.774	0.996	0.966	1.026
TSH	0.13	12.012	1	0.001	1.129	1.058	1.226
Fating blood glucose	−0.007	0.003	1	0.957	0.993	0.778	1.269
SBP	0.021	5.726	1	0.017	1.021	1.004	1.039
HAMD	0.086	5.724	1	0.017	1.089	1.016	1.169
HAMA	0.257	68.089	1	<0.001	1.293	1.217	1.375
PANSS	−0.03	2.355	1	0.125	97	0.934	1.008

MDD, major depressive disorder; TSH, thyroid stimulating hormone; SBP, systolic blood pressure; HAMD, Hamilton Rating Scale for Depression; HAMA, Hamilton Anxiety Scale; PANSS, Positive and Negative Syndrome Scale.

## 4. Discussion

To our knowledge, this is the first study to examine differences in the prevalence of suicide attempts and associated risk factors in Chinese MDD outpatients based on their age of onset. Our main findings were: (1) no significant difference was found that the prevalence of suicide attempts in early-onset MDD patients was not higher than that in late-onset MDD patients, (2) for both early- and late-onset MDD patients, patients with comorbid suicide attempts had higher HAMD score, HAMA score, PNASS score, the incidence of psychotic symptoms, as well as higher levels of TSH and blood glucose, and higher blood pressure, (3) for early-onset MDD patients, risk factors for comorbid suicide attempts were longer disease duration, higher TSH levels and anxiety symptoms; and (4) for late-onset MDD patients, risk factors for suicide attempts were higher TSH levels, SBP, HAMA score, and HAMD score.

Our study showed that no significant difference was found in the prevalence of suicide attempts in late-onset and early-onset MDD patients, which is inconsistent with previous studies, since most previous studies have shown that early-onset MDD is a distinct subtype with greater symptom severity and a higher incidence of suicidal ideation. For example, Xiao et al. found that MDD patients with earlier onset age (18–44 years) had greater comorbidity of suicidal ideation compared with those with later onset age (60–85 years) (15). Herzog et al. reported similar results, showing that early-onset MDD patients reported suicidal ideation more frequently compared with intermediate-onset and late-onset patients (18). The study by Sung et al. also showed that MDD patients with early-onset age (age < 18 years) had a higher prevalence of suicidal behavior and psychotic symptoms (27). Another study found higher levels of suicidal thoughts and sleep disturbances in both early-onset (<30 years) and late-onset (>50 years) MDD patients compared with those with intermediate onset (30–49.9 years) (28). Patients recruited in our study had not been previously treated with antidepressants, which may affect suicide attempt. Whereas the above studies have different inclusion criteria. Other factors, such as differences in clinical characteristics, data collection, and measurement instruments, could also explain these inconsistent results in different studies. At the same time a uniform definition is necessary for future in-depth studies on the onset of age-related diseases and mechanisms.

Another major finding of this study was that some clinical variables associated with suicide attempts in MDD patients were the same and some are different between early-onset and late-onset subgroups. In early-onset patients, three clinical and metabolic variables (including longer duration of illness, higher TSH levels, and HAMA score) were significantly associated with suicide attempts in MDD patients, whereas in late-onset patients, four clinical and metabolic variables, including higher TSH levels, SBP, HAMA and HAMD scores, were significantly associated with suicide attempts in MDD patients. There are many studies on risk factors for suicide attempts in MDD patients, with mixed results. For example, Fang et al. reported that total duration of disease was a risk factor for suicidal ideation in a Chinese MDD patients (29). Liang et al. indicated that a higher number of depressive episodes increase the risk of suicide attempts in patients with MDD (30). A database study of 13 major psychiatric hospitals or psychiatric departments of general hospitals in China showed that previous suicide attempts and depressive episodes with melancholic features were independently associated with recent suicide attempts in patients with MDD (31). The difference of risk factors for suicide attempts in MDD patients may be due to differences in inclusion exclusion criteria, the parameters included and the statistical strategy. The finding of our study can only represent those first-episode, untreated Chinese MDD patients, and the generalization of our results to other populations requires careful interpretation.

Our study is the first to compare suicide attempts in untreated and first-episode MDD patients at different ages of onset. We demonstrated that TSH was associated with suicide attempts in both subgroups, but we only found a significant association for the duration of disease in late-onset MDD patients, but not in early-onset MDD patients. We hypothesize that the longer duration of illness may be physically and mentally disruptive to patients and a cause of suicide attempts. Long-term disease or duration of untreated depression was associated with worse outcomes (32). However, this is only our speculation, and further investigation of this factor may help guide future treatment.

It is important to note some limitations of this study. Firstly, a causal relationship between suicide attempts and onset age and associated risk factors cannot be inferred, as this is a cross-sectional study design. Secondly, in this study, our MDD patients are from the clinical setting of the psychiatric outpatient clinic of a large general hospital, which may lead to selection bias and limit generalization to the broader Chinese population. In addition, the patients in our study were first-episode MDD patients who had not yet received medication, so the results may not be generalized to other MDD patients, such as inpatients or community patients. Thirdly, multiple variables that may also contribute to suicide attempts, such as environment, job, income, relationship with family members, and type of treatment, were not collected. Also, many antipsychotics can affect the incidence of suicide attempt. However we did not provide detailed information about antipsychotics (such as name, dosages and duration). Fortunately, as we only include patients of first episode MDD patients, so the results of this study may not be significantly affected. Finally, we did not adopt a structured assessment tool to define suicide attempts. In future studies, we will use more convincing tools to compensate for the methodological limitation of this study. Therefore, due to methodological limitations, our findings should be interpreted with caution.

In conclusion, the prevalence of suicide attempts was not higher in patients with early-onset MDD than in those with late-onset MDD. Considering the high rates of suicide attempts in both onset age groups, regular screening of suicide attempters and related psychoeducation should become a routine practice of outpatient care in general hospitals, with particular attention to outpatients aged ≥22 years. Further, the main findings of this study have significant clinical importance. A longitudinal observation of the TSH and HAMA in both early and late onset MDD will provide the necessary help to prevent suicide. And it is time to work on finding a well-rounded severity index for suicide of MDD, which can reflect the main symptoms accurately. Our study focused on the suicide attempts in early- and late-onset MDD in the Chinese Han population to minimize the potential confounding effects. Therefore, for the above two specific groups, our results are more likely to be accurate in clinical practice. It may be meaningful to predict the severity of suicidality in MDD at different ages of onset, and allow for the conduction of appropriate evidence-based clinical prevention or intervention. However, due to the limitations of our study, such as cross-sectional design, outpatients only, and lack of some factors possibly associated with suicide attempts, our findings should be considered preliminary, which should be confirmed in a longitudinal study with a large sample in the future.

## Data availability statement

The raw data supporting the conclusions of this article will be made available by the authors, without undue reservation.

## Ethics statement

The study was approved by the Institutional Review Board of the First Hospital of Shanxi Medical University (no. 2016-Y27). The patients/participants provided their written informed consent to participate in this study.

## Author contributions

XZ designed the study. XH and YS collected the data and performed the analyses. XH wrote the first draft of the manuscript. XZ and AW provided language help and writing assistance. All authors contributed to the article and approved the submitted version.

## Conflict of interest

The authors declare that the research was conducted in the absence of any commercial or financial relationships that could be construed as a potential conflict of interest.

## Publisher’s note

All claims expressed in this article are solely those of the authors and do not necessarily represent those of their affiliated organizations, or those of the publisher, the editors and the reviewers. Any product that may be evaluated in this article, or claim that may be made by its manufacturer, is not guaranteed or endorsed by the publisher.
